# Prevalence, Virulence, and Pathogenic Mechanisms of Mastitis-Associated *Klebsiella pneumoniae* in Herds and Phage-Based Control Strategies

**DOI:** 10.3390/vetsci13040352

**Published:** 2026-04-03

**Authors:** Wenhui Li, Jianwei Wang, Yangsen Wang, Pu Yan, Zhihua Ren, Tong Fu

**Affiliations:** 1 College of Veterinary Medicine, Henan Agricultural University, Zhengzhou 450046, China; liwenhui0806@stu.henau.edu.cn (W.L.); jianwei_wang2503@stu.henau.edu.cn (J.W.); wys0929@stu.henau.edu.cn (Y.W.); yanpu1158@stu.henau.edu.cn (P.Y.); 2 Ministry of Education Key Laboratory for Animal Pathogens and Biosafety, Zhengzhou 450046, China; 3 College of Animal Science and Technology, Henan Agricultural University, Zhengzhou 450046, China

**Keywords:** bovine mastitis, *Klebsiella pneumoniae*, prevalence, virulence genes, pathogenicity, antibiotic resistance

## Abstract

*Klebsiella pneumoniae* (*K. pneumoniae*) is an opportunistic and environmental mastitis pathogen prevalent in dairy herds worldwide. Owing to their genetic and genomic diversity, *K. pneumoniae* strains associated with bovine mastitis exhibit significant variation in virulence. Certain types of mastitis-causing *K. pneumoniae* strains exhibit enhanced pathogenicity and mammary adaptability, posing a serious threat to global public health. Bovine mastitis-causing *K. pneumoniae* strains can cause ultrastructural damage to bovine mammary epithelial cells (bMECs), leading to inflammatory injury, oxidative damage, apoptosis, pyroptosis, and immune evasion in bMECs. In this review, we summarize the prevalence, virulence genes, and pathogenic mechanisms of *K. pneumoniae* strains related to bovine mastitis. Given the increasing multidrug resistance of *K. pneumoniae*, we also outline the methods and mechanisms of phage therapy for *K. pneumoniae* infections, as well as future directions for treatment and prevention. These findings contribute to a deeper understanding of the population structure of mastitis-associated *K. pneumoniae* and provide valuable insights for future research on pathogenic mechanisms, vaccine development, and control strategies.

## Simple Summary

This review focuses on *Klebsiella pneumoniae*, an environmental pathogen associated with bovine mastitis. This bacterium shows high genetic diversity, and some highly pathogenic strains not only harm dairy cattle but also threaten public health. The growing problem of antibiotic resistance has further complicated its treatment. This review summarizes the prevalence and pathogenicity of *Klebsiella pneumoniae* in bovine mastitis and explains how it damages mammary cells by inducing oxidative stress, cell death, and immune evasion. In view of the challenge of drug resistance, we also discuss the potential of phage therapy as a precise antibacterial approach and propose future control strategies. Overall, this review improves our understanding of this pathogen and supports the development of vaccines and effective control measures, thereby helping to protect dairy cow health, ensure stable milk production, reduce antibiotic use, and safeguard public health by limiting zoonotic transmission.

## 1. Introduction

Mastitis is an inflammatory response in mammary tissue caused by multiple factors. As one of the most prevalent diseases in dairy production, mastitis leads to reduced productivity due to high treatment costs, resulting in approximately $35 billion in annual economic losses globally [1,2,3].

On the basis of the clinical manifestations observed in infected animals, mastitis can be classified into two primary forms: clinical mastitis (CM) and subclinical mastitis (SCM) [4]. CM typically manifests acutely. Characteristic symptoms include systemic signs (such as lethargy, anorexia, fever, and, in severe cases, mortality) as well as local symptoms (including teat redness and swelling and abnormal milk with clots, discoloration, or a watery consistency). SCM does not present visible udder abnormalities or systemic symptoms but typically results in decreased milk yield, reduced milk quality, and elevated somatic cell counts. Epidemiological data indicate a global prevalence of approximately 42% for SCM and 15% for CM [5]. In leading dairy-producing nations, including the United States, the United Kingdom, New Zealand, and Canada, the annual incidence of CM is reported to range from 13% to 40% [6]. Within Chinese dairy cattle populations, the monthly incidence of CM varies between 0.6% and 18.2%, with associated treatment costs estimated at approximately 29–135 per affected cow annually [7].

The pathogenesis of mastitis involves complex etiological factors, which can be categorized into intrinsic and extrinsic determinants. Intrinsic factors include gastrointestinal-derived mastitis resulting from dysbiosis of the animal’s intestinal/ruminal microbiota, whereas extrinsic factors involve primarily mammary infections caused by pathogenic microorganisms [8,9]. Mastitis triggered by environmental pathogens is termed environmental mastitis. The major causative agents of bovine environmental mastitis include *Escherichia coli* (*E. coli*) and *Klebsiella pneumoniae* (*K. pneumoniae*) [10,11]. The clinical symptoms of mastitis in cows caused by *K. pneumoniae* are more severe than those caused by *E. coli* [12].

*Klebsiella* is a significant genus of Gram-negative bacilli within the Enterobacteriaceae family and is commonly recognized as an opportunistic pathogen in clinical settings. Among them, *K. pneumoniae* exhibits the strongest pathogenicity toward humans. In veterinary medicine, analysis of *Klebsiella* infection cases indicates that dogs (393/697 = 56.4%), cattle (149/697 = 21.4%), and horses (98/697 = 14.1%) are the three most frequently affected animals, leading to urinary tract infections, mastitis, and reproductive disorders, respectively [13]. *K. pneumoniae* is a major opportunistic pathogen within the *Klebsiella* genus and poses a serious threat to global public health [14,15]. On dairy farms, *K. pneumoniae* is present in the teat skin, bedding materials, and fecal contaminants of cattle, leading to bovine mastitis through intramammary infection (IMI) [16].

The genome of *K. pneumoniae* comprises two primary components: the core genome and the accessory genome. The core genome has been highly conserved throughout genetic evolution, whereas the accessory genome, located on plasmids and chromosomes, has exhibited considerable complexity and diversity [17,18]. The accessory genome encodes protein products involved in the uptake of essential nutrients, such as those involved in nitrogen fixation and iron acquisition. It also encodes various virulence factors and antibiotic resistance enzymes, contributing to the diverse virulence and antibiotic resistance profiles observed among *K. pneumoniae* strains [18,19]. *K. pneumoniae* strains are classified into classical *K. pneumoniae* (cKp) and hypervirulent *K. pneumoniae* (hvKp) strains on the basis of their virulence [20,21]. Further research on the population structure of *K. pneumoniae* has revealed that the *K. pneumoniae* population encompasses multiple species and subspecies, collectively referred to as the *K. pneumoniae* species complex (KpSC) [22].

HvKp is more pathogenic than cKp. This enhanced virulence primarily manifests through two mechanisms: a thicker capsule and increased iron acquisition capacity [23]. Most hvKp strains carry core virulence factors, such as K1 and K2 capsules and O1/O2 lipopolysaccharides (LPSs). They also possess accessory virulence genes such as *rmpA/rmpA2* and *iuc*. These genes encode proteins responsible for producing hypermucoid polymers and aerobactin siderophores, respectively [24,25,26]. Under laboratory conditions, hvKp often presents a hypermucoviscous (HVM) phenotype [27]. The increasing virulence of *K. pneumoniae* strains increases disease incidence in dairy herds. This trend undoubtedly complicates the treatment of *K. pneumoniae*-induced bovine mastitis.

Research on bovine mastitis caused by *K. pneumoniae* started relatively late and lacks systematic studies. On the basis of recent global research papers on bovine mastitis and by incorporating studies on the pathological mechanisms of *K. pneumoniae* in humans and mice, this article reviews the epidemiology, virulence, and pathogenic mechanisms of *K. pneumoniae* in bovine mastitis, aiming to provide directions for future research on prevention and control.

## 2. Information Sources and Search Strategy

The research documents analyzed in this study were obtained from three electronic databases, including Web of Science, Elsevier Scopus, and PubMed. The keyword combinations used in this search include: “*Klebsiella pneumoniae*” or “*Klebsiella* spp.” and “Bovine mastitis” or “Dairy cow” and “Epidemiology” or “Prevalence” and “Drug resistance” or “Antibiotic resistance” and “Virulence genes” and “Pathogenicity” or “Bacteriophage” and “Bovine mastitis vaccines” and “Chinese herbal medicine” and “Probiotics” and “Antimicrobial peptides”. A total of 325 relevant publications were initially retrieved based on the keywords. We excluded duplicate publications, studies with clearly irrelevant research topics, and those with major flaws in research design. The remaining publications were evaluated through full-text manual reading. Finally, a total of 173 publications were included in this review for analysis and discussion.

## 3. Global Prevalence of Mastitis Caused by *K. pneumoniae*

*Klebsiella* (primarily *K. pneumoniae*) is a classic environmental pathogen found in contaminated bedding, feces, feed, air, milking equipment, and animal mucosa. Owing to variations in legislation, veterinary and laboratory services, and farmer management practices across different countries, the prevalence of bovine mastitis caused by *K. pneumoniae* varies considerably across nations and geographical regions.

There is a higher prevalence of *K*. *pneumoniae* in dairy cattle in developing countries. In Asian cattle herds, the prevalence of *K. pneumoniae* generally ranges from 1.04% to 35.91% [16,28,29,30,31,32,33,34,35,36,37,38,39,40,41]. In African cattle herds, the prevalence of *K. pneumoniae* is 3.43% to 20.70% [42,43,44,45]. The high prevalence of *K*. *pneumoniae* in bovine mastitis in developing countries is attributed primarily to poor hygienic conditions in farming environments (such as irregular bedding management), inadequate implementation of prevention and control measures, and underdeveloped pathogen surveillance systems.

The prevalence is generally below 7% in Europe [46], the Americas [47,48,49,50,51,52,53,54], and Oceania [55]. The lower prevalence is attributed to standardized intensive farming management, strict milking hygiene procedures (such as teat dipping and equipment disinfection), and a well-established surveillance network for mastitis pathogens [56].

## 4. Genetic Diversity and Molecular Epidemiology of Mastitis Caused by *K. pneumoniae*

Genotyping serves as a critical tool for investigating the epidemiology of *K. pneumoniae* in bovine mastitis. Multiple molecular techniques are currently available to characterize and type mastitis isolates, thereby revealing their genetic diversity. Genotyping methods for bovine mastitis-associated *K. pneumoniae* include ribotyping (16S amplicon sequencing), capsular genotyping, repetitive DNA sequence-based PCR (rep-PCR), random amplified polymorphic DNA (RAPD) analysis, multilocus sequence typing (MLST), and whole-genome sequencing-based single-nucleotide polymorphism (WGS-SNP) analysis [33,57,58,59]. The core objective of bacterial typing methods is to achieve precise strain identification, trace evolutionary and transmission pathways, and monitor epidemic trends by analyzing the genetic characteristics of pathogenic bacteria, thereby providing a scientific basis for disease surveillance and targeted prevention and control. The global typing data for *K. pneumoniae* in bovine mastitis are summarized in Table 1.

The string test initially served for phenotypic identification of *K. pneumoniae* isolates, enabling the differentiation of HVM *K. pneumoniae* strains [65]. RAPD type A represented the dominant genotype during a mastitis outbreak on a New York dairy farm in 2007 [58].

The rep-PCR genotyping method amplifies repetitive sequences in bacterial genomes for rapid strain identification. Rep-PCR type 1 emerged as the most prevalent type across six dairy farms in four U.S. states (Indiana, Minnesota, Wisconsin, and Pennsylvania) [58]. Rep-PCR types 9 and 17 were identified on two Chinese dairy farms, and type 17 demonstrated enhanced udder adaptability in specific farm environments [59].

MLST genotyping plays a crucial role in the prevention and control of clinical infections caused by *K. pneumoniae* and in monitoring the transmission of antimicrobial resistance. The prevalent STs in the United States include ST34, ST35, ST37, ST43, ST65, ST107, ST133, ST290, ST294, ST309, and ST791, revealing no distinct clustering between human and bovine isolates [59]. MLST identified hypervirulent ST25, ST37 and ST889 *K. pneumoniae* strains isolated from bovine mastitis samples. This presence increases the risk of cross-species *K. pneumoniae* infection [64]. *K. pneumoniae* exhibits genomic diversity, and the prevalent STs vary across dairy farms in different regions. ST107 and ST43 have been frequently detected in China [32,34], indicating that these two clones represent globally dominant strains. China-specific ST896 is closely related to the U.S. strains ST230 and ST37 [32]. ST37 has been reported in humans, chickens, cattle [66], and companion animals [67]. While ST11, ST258, and ST15 are the major global CRKP clones in humans, ST11 and ST258 have been detected in cattle as well [32,68]. These findings indicate that the emergence of carbapenemase-producing bacteria has become a global public health issue and that their presence in animals (including livestock), wildlife, and the environment further complicates the global landscape of antimicrobial resistance.

WGS enables the identification of *K. pneumoniae* subtypes and provides detailed information on their virulence and antimicrobial resistance genes, thereby offering direct evidence for assessing transmission risk and guiding targeted treatment. WGS-SNP classified bovine mastitis-causing *K. pneumoniae* strains into three phylogenetic groups: KpI (*K. pneumoniae*), KpII (*K. quasipneumoniae*) and KpIII (*K. variicola*) [14,22,69,70]. This classification is based on molecular typing techniques, such as the analysis of chromosomal β-lactamase genes (e.g., blaSHV and *blaOKP*) and their flanking sequences. The distinct core resistance genes carried by KpI, KpII, and KpIII serve as differentiating markers [71].

According to a study by Kajal Mishra et al., KpIII accounted for 14% of clinical isolates, primarily from urine (52.3%), whereas KpII constituted 24%, with a higher proportion in pus samples (36.1%). KpI demonstrated the highest isolation rate overall, representing 93.62% of all the samples. KpI was identified as the dominant group responsible for *K. pneumoniae* mastitis in dairy cattle [22,31]. In terms of antimicrobial resistance, KpIII demonstrates significant multidrug resistance, with a markedly higher resistance rate to polymyxin B (42.85%) than to KpI (11.82%). Resistant strains frequently exhibit nonsynonymous mutations in the PhoP/PhoQ two-component regulatory system. Both KpI and KpII also commonly carry β-lactam resistance genes (e.g., *blaNDM*, *blaOXA-48*, and *blaSHV*), although their specific resistance profiles require analysis in conjunction with the clinical context. Additionally, KpIII is strongly associated with severe infections (such as in ICU patients) [71], suggesting potentially increased pathogenicity or adaptability. However, these data are derived primarily from human infection studies and require further validation in bovine populations.

## 5. Damage Effects on the Organism

After infecting bovine mammary epithelial cells (bMECs), *K. pneumoniae* induces a series of time-dependent structural damage through adhesion and invasion. The activation of pattern recognition receptors in the host innate immune system triggers inflammatory responses, promotes the release of proinflammatory factors, and leads to mammary tissue congestion and edema. Infection also causes oxidative damage, apoptosis, and potential pyroptosis in bMECs, collectively leading to epithelial cell death and mammary dysfunction.

### 5.1. Effects of Kp on the Structure of bMECs

**Figure 1:**
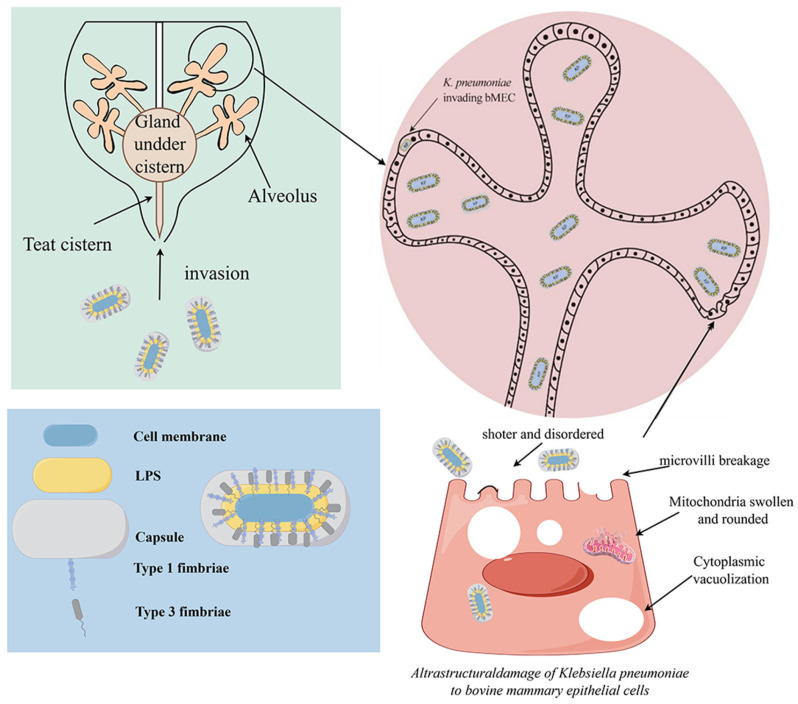
The process of *K. pneumoniae* infection in the mammary gland and its damaging effects on bMECs. *K. pneumoniae* originating from the farm environment (e.g., bedding, fencing, and teat skin) ascends through the teat orifice and canal into the gland cistern. The bacteria subsequently adhere to and colonize the surface of bMECs, followed by invasion, leading to disruption of the cellular ultrastructure. Created with Figdraw.com. Author: [Wenhui Li], (accessed on 11 January 2026).

As frontline defenders, bMECs play a critical role in counteracting pathogenic infection [72]. *K. pneumoniae* can enter mammary tissue through the teat canal at the distal end of the cow’s teat, adhere to and invade mammary epithelial cells, causing cellular damage (such as cellular swelling, nuclear pyknosis, and necrosis), and this damage exhibits a time-dependent pattern [73]. In vitro infection models have demonstrated that *K. pneumoniae* infection of bMECs increases the release of lactate dehydrogenase (LDH), leading to ultrastructural damage in bMECs and impairing the morphology and function of the cell membrane, cytoplasm, and organelles. The integrity of the cell membrane is compromised, with pores and vacuoles forming on the surface, along with ruptured microvilli; the cytoplasm shows vacuolation; the endoplasmic reticulum is swollen and vacuolated; and the mitochondria swell and gradually become rounded, with their matrix compressed and dense, the crista gaps widened, and cavities of various sizes formed. Activation of FNIP1 leads to a decrease in ATP, downregulation of the mitochondrial membrane potential, and increased mitochondrial permeability, resulting in mitochondrial damage and reduced synthesis of milk fat and proteins [74]. The process of *K. pneumoniae* mammary tissue infection and its impact on bMEC architecture are summarized in Figure 1.

### 5.2. Inflammatory Response

**Figure 2:**
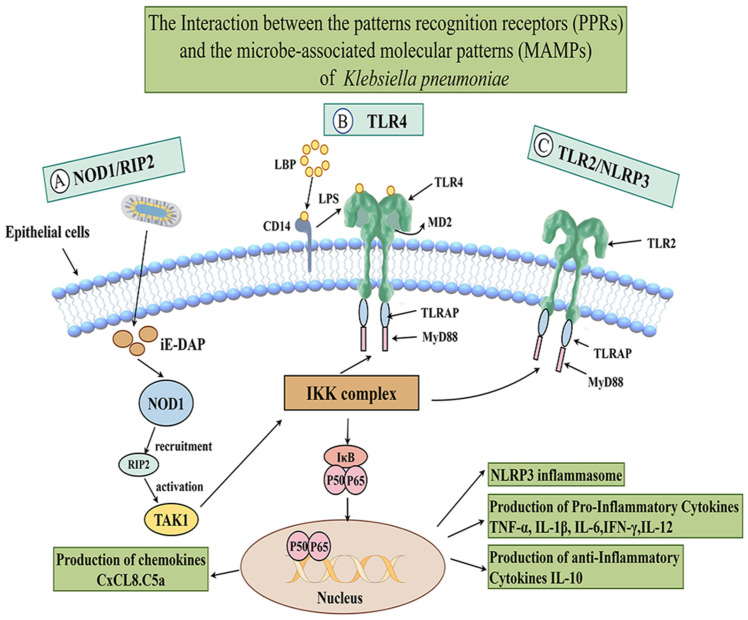
Interactions between pattern recognition receptors (PPRs) and microbe-associated molecular patterns (MAMPs) of *K. pneumoniae*. Infection of the mammary gland by *K. pneumoniae* is accompanied by an inflammatory response, in which the pattern recognition receptors NOD1, TLR2 and TLR4 mediate signal transduction and collectively induce the production of cytokines and chemokines via the NF-κB signaling pathway. Created with Figdraw.com. Author: [Wenhui Li], (accessed on 30 December 2025).

Infection of the mammary gland by *K. pneumoniae* triggers inflammatory damage, characterized by an influx of polymorphonuclear neutrophils (PMNs) and a consequent increase in the somatic cell count (SCC). The interaction between *K. pneumoniae* and the host innate immune system begins when pattern recognition receptors (PRRs), notably NOD1, TLR2 and TLR4, recognize bacterial components. This recognition activates multiple signal transduction pathways that converge on the downstream NF-κB signaling pathway. The activation of NF-κB promotes the expression of proinflammatory cytokines, initiating a potent inflammatory response. This cascade ultimately results in severe pathological manifestations in mammary tissue, including hyperemia, edema, and milk stasis [73,75]. The interactions between pattern recognition receptors (PPRs) and microbe-associated molecular patterns (MAMPs) of *K. pneumoniae* are summarized in Figure 2.

NLRs bind to specific stimuli from invading cells, followed by their own oligomerization. These proteins subsequently undergo homotypic interactions with the CARD domain-containing RIP2 protein via their CARD domains. RIP2 then binds to the IκB kinase (IKK) complex, thereby activating the intracellular NF-κB pathway and inducing cytokine expression [76].

Toll-like receptor 2 (TLR2), located on the cell surface, participates in the immune response of bMECs by regulating the release of cytokines such as TNF-α, IL-1β, IL-6, and IL-10, thereby triggering an inflammatory response [77]. During *K. pneumoniae* infection, the key adapter molecules connecting TLR2 recognition of its lipopeptide Pam3CSK4 to the induction of proinflammatory effects are TIRAP and MyD88 [78,79,80].

Toll-like receptor 4 (TLR4) on the cell surface also induces an inflammatory response in bMECs through the TLR4/NF-κB signaling pathway [81,82].

### 5.3. Oxidative Damage

*K. pneumoniae* infection of the mammary gland induces cellular oxidative damage. *K. pneumoniae* isolated from CM samples stimulated bMECs to significantly increase ROS and malondialdehyde (MDA) levels while markedly reducing total antioxidant capacity (T-AOC) at 3, 6, and 12 h post-infection (pi) [83]. *K. pneumoniae* can interfere with the Nrf2/xCT/GPX4 pathway, leading to mitochondrial dysfunction, accumulation of reactive oxygen species (ROS), and ultimately triggering cellular ferroptosis [84]. *K. pneumoniae* triggers ferroptosis in bMECs via NCOA4-mediated ferritinophagy. This process increases intracellular Fe^2+^ levels, resulting in the collapse of the mitochondrial membrane potential and accumulation of lipid peroxides, ultimately contributing to epithelial cell damage and lactation dysfunction [85]. When bMECs undergo ferroptosis induced by *K. pneumoniae*, selenium [11,86] can exert an antagonistic effect, while ferrostatin-1 (Fer-1) can alleviate both the inflammatory response and ferroptosis.

### 5.4. Apoptosis

Apoptosis is a regulated form of cell death involved in various normal physiological processes [87]. During bMECs infection, *K. pneumoniae* activates the intrinsic mitochondrial pathway, leading to apoptosis. In vivo studies have shown that 2 h after bMECs are infected with *Klebsiella pneumoniae*, the mRNA expression levels of the proapoptotic proteins caspase-3 and caspase-9 and the intermembrane space protein cytochrome c (cyt-c) are elevated, accompanied by a significant decrease in the mitochondrial membrane potential (MMP), indicating that *K. pneumoniae* induces excessive apoptosis in bMECs [83,88].

After *Klebsiella pneumoniae* invades host cells, enterobactin induces mitochondrial dysfunction and reactive oxygen species (ROS) accumulation by chelating intracellular iron ions, thereby activating the PINK1/Parkin- and BNIP3-dependent mitophagy pathways [89].

### 5.5. Pyroptosis

**Figure 3:**
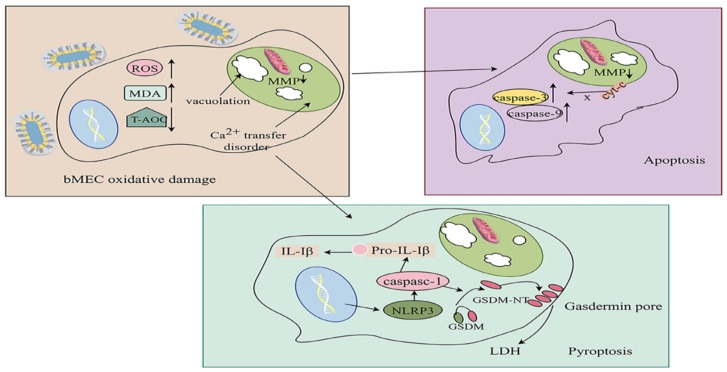
*K. pneumoniae*-induced apoptosis, pyroptosis, and oxidative damage in bMECs. Following *K. pneumoniae* infection of the mammary gland, bMECs undergo oxidative stress, leading to increased reactive oxygen species (ROS) levels and subsequent oxidative damage. This adversely affects mitochondrial structure and function: structural alterations include mitochondrial vacuolization and severe crista degeneration, whereas functional impairment manifests as Ca^2+^ dyshomeostasis. Oxidative damage further promotes the release of cytochrome c (cyt-c) from mitochondria into the cytoplasm and increases the expression of proapoptotic proteins, thereby triggering apoptosis. In addition, lactate dehydrogenase (LDH) release into the extracellular space may be associated with *K. pneumoniae*-induced pyroptosis during infection. Created with Figdraw.com. Author: [Pu Yan], (accessed on 11 January 2026).

Pyroptosis is an inflammatory form of programmed cell death (PCD) mediated by inflammatory caspases (caspase-1, -4, -5, and -11) [90]. This process involves the activation of inflammasomes and caspases [91]. Activated caspase-1 cleaves gasdermin proteins (GSDMs), releasing their N-terminal domains (GSDM-NTs), which oligomerize and form pores in the plasma membrane, leading to lytic cell death [92,93]. Pyroptosis is characterized by cell swelling, the release of cellular contents, and membrane rupture, accompanied by the secretion of proinflammatory cytokines such as IL-1β and IL-18, thereby amplifying the inflammatory response [94,95]. Infection of female C57BL/6J mice (6–8 weeks old) with 1 × 107 CFU of Kp/mouse induces pyroptosis in alveolar macrophages (AMs). The mechanisms underlying oxidative damage, apoptosis, and pyroptosis in bMECs following Kp infection are illustrated in Figure 3.

## 6. Main Virulence Factors of *K. pneumoniae*

Genotyping of *K. pneumoniae* strains isolated from bovine mastitis reveals significant genetic diversity. The pathogenicity of *K. pneumoniae* is dependent primarily on four major virulence factors, namely, fimbriae (pili), capsules, lipopolysaccharides (LPS), and siderophores, which are crucial for bacterial colonization, growth within host tissues, and evasion of host immune clearance.

### 6.1. Capsules

The capsule, a polysaccharide matrix that coats the cell, is one of the most important virulence factors of *K. pneumoniae*, enabling the bacterium to evade recognition by the host immune system through mechanisms such as antiphagocytosis, suppression of early inflammatory responses, neutralization of antimicrobial peptides to reduce immune reactions, and inhibition of dendritic cell maturation [96,97,98]. In both classical *K. pneumoniae* and hypervirulent *K. pneumoniae* (hvKP) strains, the primary genes responsible for capsule synthesis are located within the cps gene cluster, including genes such as *wzi*, *wza*, *wzb*, *wzc*, *gnd*, *wca*, *cpsB*, *cpsG*, and *galF* [99]. *RmpA*/*rmpA2* are mucoid phenotype regulatory genes that control mucus viscosity [25]. The hypermucoviscous phenotype (HVM) is typically observed in hvKP [27]. On the basis of differences in their capsular polysaccharide antigens, *K. pneumoniae* can be classified into 82K serotypes, which originate from 79 distinct capsular structures [100]. Studies from multiple countries worldwide have shown that *K. pneumoniae* causes bovine mastitis via multiple serotypes, including K1, K2, K3, K5, K54, and K57, which are recognized as zoonotic serotypes [22,36,59,64]. In Egypt, the K1 and K2 serotypes of *K. pneumoniae* are prevalent among dairy cattle with mastitis and buffalo herds [42]. Among *K. pneumoniae* strains isolated from bovine mastitis in China, the predominant capsular type is K57 (in CM samples), followed by K1, K5, and K54 (in bulk tank milk) [34,101]. In the United States, the predominant capsular type among *K. pneumoniae* strains isolated from bovine mastitis is K101 (primarily from Iowa), followed by K107 [22]. In the Peshawar region of Pakistan, K2 is the most common capsular type among *K. pneumoniae* strains isolated from bovine mastitis, followed by strains of K1, K5, and K54 [36].

*K. pneumoniae* strains can be classified into cKPs and hvKPs on the basis of their degree of virulence [20,21]. Compared with cKPs, hvKPs exhibit greater pathogenicity through enhanced self-defense mechanisms, such as thickened capsules, hypermucoviscosity, and enhanced iron uptake [23]. Among these, serotypes such as K1, K2, K5, K54, and K57 are closely associated with highly virulent strains, with K1 and K2 generally exhibiting hypervirulent and hypermucoviscous phenotypes [42]. Compared with K1/K2 strains, K57 strains exhibit greater serum tolerance and biofilm-forming ability but relatively weaker resistance to neutrophil phagocytosis, which is associated with the capsular polysaccharide content [102].

### 6.2. Fimbriae

Bacterial surface fimbria serve as crucial mediators for adhesion to host cells. *K. pneumoniae* possesses two primary types—type I and type III fimbriae—which facilitate bacterial attachment to host cell surfaces, marking the initial step of Kp infection and colonization in the host. Type I fimbriae are filamentous projections on the bacterial cell surface and are encoded by the *fimABCDEFGHK* gene cluster. These fimbriae carry the tip adhesin FimH, which binds to mannose-containing glycoproteins on host cells, thereby mediating the colonization of *K. pneumoniae* on epithelial tissues [103]. In mouse models generated from human-derived *K. pneumoniae*, the *fimK* gene encodes FimK, which enhances *K. pneumoniae* serum resistance, capsule production, and resistance to phagocytosis [104].

Type III fimbriae are encoded by the *mrkABCDF* gene cluster, exhibit a helical structure, and are composed of the major subunit *mrkA* and the adhesin protein *mrkD*. Type III fimbriae promote adhesion to abiotic surfaces and biofilm formation across strains from diverse sources [105,106].

In studies on human-derived clinical *K. pneumoniae* [107] and on bovine-derived *K. pneumoniae* [34,108], both type I and type III fimbriae were found to promote biofilm formation. Biofilms affect the development of drug resistance in *K. pneumoniae* [109], and the strength of biofilms in bovine-derived *K. pneumoniae* shows a positive correlation with drug resistance [110]. In isolates from clinical and subclinical mastitis patients, the detection rates of the *fimH*, *mrkA*, and *mrkD* genes exceed 80% [101]. Given that *fimH* and *mrkA* are involved in fimbrial adhesion during the early stages of biofilm formation [111,112], their dominant detection in bovine-derived isolates suggests that fimbriae may play a significant role in intramammary infection and colonization, warranting further investigation. However, most biofilm experiments have been conducted in vitro. However, further studies are still needed to understand the actual relevance of these findings to intramammary biofilm behavior and in vivo pathogenicity.

### 6.3. Siderophores

Siderophores are recognized as important virulence factors of *K. pneumoniae*. Iron is an essential metal ion for bacterial growth, yet free iron (Fe^3+^) is scarce under physiological conditions because it binds to transferrin and heme during infection. Therefore, the production of high-affinity siderophores by *K. pneumoniae* to acquire iron is essential for its survival and replication. The siderophores secreted by *K. pneumoniae* primarily include enterobactin (Ent), aerobactin (Aer), salmochelin (Sal), and yersiniabactin (Ybt). Enterobactin is present in both cKP and hvKP [99], making it the primary iron-uptake system for *K. pneumoniae* and the most common siderophore in bovine strains [59]. The biosynthesis and transport of enterobactin are encoded by the chromosomal gene clusters *entABCDEF* and *fepABCDG* [113]. The functional unit of enterobactin is encoded by the *entB* gene [101]. The *entB* gene is widely present in clinical mastitis isolates of *K. pneumoniae* in China, and its detection rate is comparable to that of human-derived isolates [65]. Moreover, the detection rate of *entB* is significantly greater in clinical mastitis isolates than in subclinical mastitis isolates, suggesting that this gene may be associated with infection severity. After invading host cells, *K. pneumoniae* secretes siderophores (such as enterobactin), which chelate iron ions within the host cells. This leads to mitochondrial dysfunction and the accumulation of reactive oxygen species (ROS), subsequently activating the PINK1/Parkin- and BNIP3-dependent mitophagy pathways. Excessive activation of mitophagy ultimately results in apoptosis [89]. The *kfuABC* iron-acquisition system is involved in the iron uptake process of *K. pneumoniae*. *kfuABC* is more prevalent in CM isolates [59]. These findings suggest that *kfuABC* may play an important role in tissue invasion and infection. Hypervirulent *K. pneumoniae* mutants lacking the *kfu* gene fail to cause mortality in mice, highlighting the importance of enhanced iron-acquisition systems for the pathogenicity of *K. pneumoniae* [114].

The ferric citrate uptake system (fec iron acquisition system) plays a significant role in iron sequestration. The detection rate of fec genes is increased in CM strains [22]. While the ferric citrate uptake system is an important iron acquisition pathway in *E. coli*, the fec system in *K. pneumoniae* strains may have been acquired through horizontal gene transfer [115,116]. These findings indicate that bacteria are undergoing key adaptive evolution within dairy cattle populations, raising concerns about the emergence of more virulent strains and potential risks of cross-species transmission. The fec operon is frequently clustered with the lactose (lac) operon, facilitating the utilization of lactose and citrate in bovine milk [117]. This endows bovine-derived *K. pneumoniae* with a selective growth advantage, enabling faster proliferation within the bovine mammary gland.

### 6.4. Lipopolysaccharide

Lipopolysaccharide (LPS), also known as endotoxin, is a major and essential component of the outer membrane surface in all Gram-negative bacteria. The detection rates of the LPS virulence-related genes *uge* and *wabG* exceed 85% in bovine-derived *K. pneumoniae* [108]. The *uge* gene promotes lipopolysaccharide synthesis and enhances immune evasion [118]. The *wabG* gene is prevalent in clinical *K. pneumoniae* isolates [108], and strains lacking this gene fail to produce the LPS outer core or retain capsular antigens, resulting in reduced virulence [119]. LPS is composed of O-antigen, core oligosaccharide, and lipid A. On the basis of structural differences in the O-antigen, it can be classified into nine distinct serotypes, the most common of which are O1, O2, and O3 [120]. The lipid A component is the primary immunostimulatory center, serving as a ligand for the pattern recognition receptor TLR4. The O antigen intertwines with capsular fibers to stabilize the capsule and protects against complement-mediated killing by promoting C3b binding to the outer membrane, thereby preventing the formation of the membrane attack complex [121,122,123,124].

### 6.5. Variational Characteristics of Virulence Genes in K. pneumoniae Originating from Bovine Mastitis

Genomic studies have revealed the distinctive genetic makeup of *K. pneumoniae* strains isolated from bovine mastitis. In the study by Yang et al. [59], compared with human-derived strains, *K. pneumoniae* isolated from the milk of cows with CM and SCM presented a greater prevalence of genes related to metal ion (iron, zinc, and calcium) metabolic pathways. Zheng et al. [22] compared the virulence genes of *K. pneumoniae* strains isolated from dairy cows with mastitis, human patients, and dairy farm environments. They reported that the genes most closely associated with the pathogenicity of bovine-derived *K. pneumoniae* strains involved primarily ferric citrate uptake, lactose fermentation, and heavy metal resistance. Nearly all bovine mastitis isolates carry complete copper, silver, and arsenic core resistance gene clusters, whereas such clusters are present in less than 40% of environmentally derived and human-derived strains. Additionally, approximately 60% of bovine-derived *K. pneumoniae* strains contain chromate efflux transport genes, while the distribution proportion of these genes in non-bovine strains is less than 10%. These findings suggest that bovine mastitis-derived *K. pneumoniae* may serve as a microbial reservoir that carries multiple metal resistance genes. Vaidya et al. [125] demonstrated the antimicrobial efficacy of metals such as silver, copper, platinum, gold, and palladium against biofilm-forming bacteria, and the aforementioned metal resistance genes may spread among different microorganisms through horizontal gene transfer. This could promote the emergence of more widely resistant strains, posing potential risks to clinical treatment and public health.

## 7. Immune Evasion

### 7.1. Disruption of Innate Immunity for Immune Evasion

**Figure 4:**
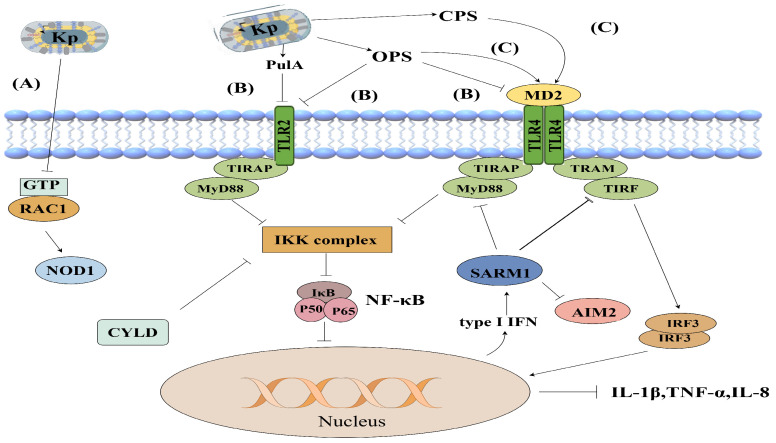
*K. pneumoniae* subverts the innate immune response in epithelial cells to suppress early inflammation and evade immune surveillance. (A) Via a NOD1-dependent pathway, *K. pneumoniae* upregulates the deubiquitinase CYLD, leading to attenuated NF-κB activation and a diminished inflammatory response. (B) The virulence factors LPS O-polysaccharide and pullulanase PulA decrease the expression of proinflammatory cytokines (IL-1β, TNF-α, and IL-8) by impairing the activation of the TLR2-TLR4-MyD88 pathway and subsequent NF-κB signaling. (C) CPS and O-polysaccharide promote SARM1 expression via IFN production induced by the TLR4-TRIF-TBK1-IRF3 signaling axis. SARM1 then negatively regulates both MyD88 and TRIF, thereby suppressing AIM2 inflammasome activation and antagonizing host immunity. Created with Figdraw.com. Author: [Yangseng Wang], (accessed on 28 December 2025).

During epithelial cell infection, *K. pneumoniae* suppresses Rac1 activation and hijacks NOD1 signaling to upregulate CYLD and MKP-1 expression. This attenuates NF-κB and MAPK-dependent signaling, reduces IL-1β and IL-8 production, and thereby exerts anti-inflammatory effects, curbing excessive inflammation in the early stages of infection [126]. The process is shown in Figure 4.

*K. pneumoniae* can also hijack proteins involved in immune homeostasis to evade host immune surveillance. SUMOylation is a posttranslational modification mediated by small ubiquitin-like modifier (SUMO) proteins [127], and it regulates various cellular processes, including innate immunity. Sentrin/SUMO-specific protease 2 (SENP2) is a deSUMOylating enzyme that reverses SUMO modification [128]. *K. pneumoniae* inhibits the NEDDylation of the Cullin-1 subunit within the E3-SCF-βTrCP complex via the deubiquitinase CSN5, thereby impairing K48-linked ubiquitination and subsequent proteasomal degradation. This leads to an increase in the level of the deSUMOylating enzyme SENP2. Moreover, *K. pneumoniae* simultaneously induces the expression of CSN5 through the EGFR-AKT-ERK-GSK3β signaling pathway, further modulating host cell protein modifications. Additionally, the bacterium induces type I interferon (IFN) production via the TLR4-TRAM-TRIF signaling pathway. IFN then mediates signal transduction through the IFNAR1 receptor, ultimately downregulating SUMOylation levels via let-7 microRNA, thereby suppressing the host immune response [129].

### 7.2. The Capsule Facilitates Immune Evasion by K. pneumoniae

*K. pneumoniae* resists phagocytosis by neutrophils through its capsule rather than by directly suppressing the function of immune cells [99]. Hypervirulent *K. pneumoniae* (HvKp) strains typically modulate capsule polysaccharide production via regulators of the mucoid phenotype (e.g., *rmpA*/*rmpA2*), resulting in a thicker capsule [23,130]. Although studies have shown that hvKp is more resistant to phagocytosis and neutrophil extracellular trap-mediated killing than cKp [131,132], such traits have not yet been reported in hvKp strains associated with bovine mastitis. Studies indicate that under in vitro skim milk medium conditions, *K. pneumoniae* isolates from chronic mastitis produce a thicker capsule and exhibit enhanced evasion of polymorphonuclear neutrophil-mediated killing [133].

### 7.3. Lipopolysaccharides Act as Key Mediators of Immune Evasion in K. pneumoniae

#### 7.3.1. Suppression of the TLR2-MyD88-NF-κB Pathway

Functional genomic screening revealed that the LPS O-polysaccharide and type 2 secretion system (T2SS)-secreted pullulanase PulA of *K. pneumoniae* disrupts TLR-mediated pathogen recognition, thereby suppressing TLR2-MyD88-NF-κB signaling pathway activation [134]. This represents a key mechanism by which *K. pneumoniae* evades host immune surveillance. The schematic of the mechanism is presented in Figure 4.

#### 7.3.2. Limited Activation of the TLR4-TRIF-IRF3 Signaling Pathway

The LPS O-polysaccharide of *K. pneumoniae* suppresses activation of the TLR4–MyD88 pathway, attenuating NF-κB activation and IL-8 secretion. Similarly, PulA interacts with glycans on the host cell surface, leading to limited activation of the TLR4–MyD88 pathway upon *K. pneumoniae* infection [134]. Moreover, *K. pneumoniae* infection activates the TLR4–TRIF–IRF3–IFNAR1 axis to modulate inflammatory responses. Through the TLR4–TRIF–IRF3 pathway, *K. pneumoniae* induces IFN production, which subsequently alters the expression of immune-related proteins and facilitates evasion of host immune surveillance [135]. The schematic of the mechanism is presented in Figure 4.

### 7.4. Immune Evasion by K. pneumoniae Is Mediated Through SARM1

Sterile alpha and TIR motif-containing protein 1 (SARM1) is an adaptor protein harboring a TIR domain that participates in regulating TLR signaling and inflammasome activation [136]. SARM1 plays a critical role in *K. pneumoniae* immune evasion strategies. Capsular polysaccharide (CPS) and LPS O-polysaccharides from *K. pneumoniae* induce SARM1 expression. Through TIR-TIR domain interactions, *K. pneumoniae* exploits SARM1 to attenuate both MyD88- and TRIF-dependent signaling, thereby modulating inflammation controlled by these adaptors [135]. Furthermore, SARM1 negatively regulates TLR signal transduction, promotes the production of the anti-inflammatory cytokine IL-10, and suppresses *K. pneumoniae*-induced AIM2 inflammasome activation, thereby limiting IL-1β production and subverting macrophage antibacterial immunity [135]. The schematic of the mechanism is presented in Figure 4.

*K. pneumoniae* is a classic environmental pathogen, with cattle manure, bedding materials (especially dried manure solids), and contaminated pastures serving as its primary reservoirs. Its transmission likely occurs through a fecal-oral route, enabling circulation and amplification within herds [137]. Consequently, controlling environmental exposure is critical for prevention, yet substantial efforts are needed to improve barn hygiene, bedding management, and premilking practices. In practical production, it is essential to implement procedures such as premilking for disinfection, drying, and postmilking for disinfection; maintain clean, dry conditions in barns, resting areas, exercise yards, and milking parlors; and ensure that bedding remains dry, clean, and regularly replaced. However, the comprehensive and consistent application of these management measures faces numerous challenges.

## 8. Antimicrobial Resistance in *K. pneumoniae* from Bovine Mastitis

The incidence of bovine mastitis, an important opportunistic pathogen, *K. pneumoniae*, has increased in recent years. The challenge of prevention and control is particularly pronounced in multidrug-resistant (MDR) and extended-spectrum β-lactamase (ESBL)-producing strains [138]. Therefore, the development of novel therapeutic strategies is urgently needed. Phage therapy, as a biocontrol agent with high specificity and a favorable safety profile, has demonstrated considerable potential in antiinfective applications, showing promising prospects, especially for controlling *K. pneumoniae* infections [139].

### Prevalence of Antimicrobial Resistance in K. pneumoniae from Bovine Mastitis

Numerous investigations have revealed the current status of antimicrobial resistance in *K. pneumoniae* strains isolated from bovine mastitis, highlighting a concern of global significance. *K. pneumoniae* of bovine mastitis origin generally exhibits high resistance rates to ampicillin, tetracycline, and sulfonamides but typically remains susceptible to fluoroquinolones (e.g., ciprofloxacin) and carbapenems (e.g., meropenem) in most regions, with correspondingly low resistance rates. However, carbapenem resistance has been reported at a low frequency (e.g., 3.8%) in certain areas, such as Xinjiang, China, warranting vigilance against the potential spread of such resistance. The detailed resistance rates for more bovine-derived *K. pneumoniae* isolates are provided in Table 2.

Bovine mastitis-associated *K. pneumoniae* strains exhibit widespread and severe multidrug resistance, including the emergence of carbapenem-resistant strains. A large-scale study covering seven Chinese provinces (Jiangsu, Anhui, Hebei, Fujian, Guangdong, Shanxi, and Zhejiang) further revealed this situation: among 108 *K. pneumoniae* isolates obtained from 763 mastitic milk samples, 49.07% demonstrated MDR. Some strains carry ESBL-producing genes (e.g., *blaNDM-5*), and even carbapenem-resistant hypervirulent *K. pneumoniae* have been identified [140]. Concurrently, among isolates from dairy farms in Jiangsu and Shandong, China, 6.06% of strains were resistant to at least three classes of antibiotics [31], which aligns with international findings. For example, two ESBL-producing strains detected across 28 Scottish dairy farms presented resistance to amoxicillin-clavulanic acid, streptomycin, tetracycline, cefotaxime, cefalexin and cefquinome [141]. *K. pneumoniae* was identified as a high-risk drug-resistant strain, with a resistance rate of 96.43% to β-lactam antibiotics and a multidrug resistance rate of 25% [142]. These findings underscore the necessity of surveillance and control of antimicrobial resistance in bovine mastitis-associated *K. pneumoniae* to curb its global dissemination.

**Table 2:** Antibiotic resistance rates of *K. pneumoniae* strains isolated from bovine mastitis.

Antibiotic Class	Specific Drug	Resistance Rate (%)	Country	Reference
β-lactams	Ampicillin	100%	China	[31]
		96.6%	Republic of Korea	[108]
	Amoxicillin	100%	China	[31]
		80.5%	Pakistan	[37]
		67.6%	China	[66]
	Piperacillin	3.03%	China	[31]
		10.3%	China	[66]
	Penicillin	85.3%	China	[66]
	Amoxicillin-Clavulanic Acid	7.1%	Scotland	[141]
		3.4%	Republic of Korea	[108]
	Cefuroxime	4.55%	China	[31]
	Cefalotin	4.55%	China	[31]
	Cefotaxime	3.03%	China	[31]
	Cefoperazone	1.52%	China	[31]
	Ceftazidime	1.52%	China	[31]
		19.44%	China	[140]
		13.8%	Republic of Korea	[108]
		6.9%	Republic of Korea	[108]
		4.63%	China	[140]
	Cefalexin	36.11%	China	[140]
	Cefazolin	31.3%	China	[35]
	Cephalothin	11.8%	China	[66]
	Ceftiofur	25.93%	China	[140]
	Cefepime	6.9%	Republic of Korea	[108]
	Cefquinome	4.8%	Scotland	[141]
	Cefalexin	4.8%	Scotland	[141]
	Cefoxitin	3.8%	China	[35]
		6.9%	Republic of Korea	[108]
	Meropenem	5.56%	China	[140]
		3.8%	China	[35]
Aminoglycosides	Gentamicin	30.56%	China	[140]
		12.12%	China	[31]
	Tobramycin	12.12%	China	[31]
	Gentamicin	8.3%	Pakistan	[37]
		5.9%	China	[66]
		3.4%	Republic of Korea	[108]
	Streptomycin	69.4%	Pakistan	[37]
		43.52%	China	[140]
		41.2%	China	[66]
		26.2%	Scotland	[141]
	Amikacin	8.3%	Pakistan	[37]
		1.85%	China	[140]
Tetracyclin	Tetracycline	39.7%	China	[35]
		34.5%	Republic of Korea	[108]
		33.33%	China	[140]
		27.85%	Pakistan	[37]
		21.21%	China	[31]
		19%	Scotland	[141]
		17.6%	China	[66]
Amphenicols	Chloramphenicol	33.3%	Pakistan	[37]
		13.64%	China	[31]
	Florfenicol	14.81%	China	[140]
Polymyxins	Polymyxin E	<10.0%	China	[35]
Combinations	Trimethoprim-Sulfmethoxazole	20.7%	Republic of Korea	[108]
		17.59%	China	[140]
Sulfonamides	Sulfamethoxazo	18.52%	China	[140]
	Sulfisoxazole	31%	Republic of Korea	[108]
Quinolones	Ciprofloxacin	<10.0%	China	[35]

## 9. Prevention and Control of Bovine Mastitis Caused by *K. pneumoniae*

Antibiotics remain the mainstay for treating bovine mastitis caused by *K. pneumoniae* infection. *K. pneumoniae* is intrinsically prone to developing antimicrobial resistance, and its resistance genes may spread among different bacterial species, even between humans and animals, via mobile genetic elements such as plasmids, posing a serious challenge at the “One Health” level [143]. Although surveillance data from some regions still indicate relatively high susceptibility of *K. pneumoniae* to key antibiotics such as cephalosporins and carbapenems [144,145], plasmids carrying carbapenemase genes (e.g., *blaNDM*) have been found to be shared among *K. pneumoniae*, *Escherichia coli*, and other species in other parts of the world and across different hosts. These plasmids may even originate from human clinical settings or the environment [143].

However, with the rise of drug-resistant strains and the continuous emergence of new resistant variants, global efforts have accelerated the development and application of alternative antibiotic strategies to address the growing therapeutic challenges.

### 9.1. Bovine Mastitis Vaccines

Vaccines represent one of the most promising tools for preventing environmental mastitis caused by *K. pneumoniae*. Variations in vaccine protective efficacy may be related to factors such as farm management practices, different infecting strains, and sample size, among others.

Genomic analysis of bovine mastitis-associated *K. pneumoniae* serotype K57 revealed that the expression levels of the adhesion genes *fimA*, *fimC*, and *fimG* are correlated with strain virulence levels [146]. Inoculation of mice with recombinant FimA, FimC, and FimG proteins derived from clinical mastitis isolates elicited a significant humoral immune response in mouse models, with FimG demonstrating promising protective effects. Thus, recombinant FimG protein vaccines show considerable potential for preventing *K. pneumoniae* mastitis [147]. However, since the study employed intraperitoneal injection to simulate infection, it did not fully replicate the natural ascending infection process via the teat canal characteristic of mastitis. Therefore, these findings require further validation in mammary tissue-specific models, such as co-culture with mammary epithelial cells or intramammary challenge. Nevertheless, this work provides strong practical evidence supporting immunization as a strategy for preventing mastitis.

### 9.2. Chinese Herbal Medicine for the Treatment of Bovine Mastitis Caused by K. pneumoniae

To address the growing challenge of antibiotic resistance, we should fully leverage the strengths of traditional Chinese medicine, which employs multiple components, targets multiple pathways, and achieves holistic regulation. Both paeonol and berberine hydrochloride exhibit inhibitory effects on *K. pneumoniae*. Paeonol [148] exerts antibacterial activity by disrupting cell membrane integrity and inhibiting bacterial adhesion and biofilm formation, whereas berberine hydrochloride [149] also has growth-inhibitory effects on carbapenem-resistant *K. pneumoniae* in vitro. Both compounds show promise as potential antimicrobial agents. Studies indicate that baicalein [150] not only acts alone against multidrug-resistant *K. pneumoniae* (MDR Kp) but also has synergistic effects when combined with meropenem or polymyxin E. Stably binding to and suppressing the expression of the quorum-sensing genes LuxS/LuxR, it effectively disrupts biofilm structure and eliminates residual bacteria, offering a novel multitarget strategy for treating bacterial pneumonia caused by MDR Kp.

### 9.3. K. pneumoniae Bacteriophages

Bacteriophages (phages) are viruses that infect bacteria, fungi, and actinomycetes. Owing to their high specificity and capacity for self-replication, these compounds are promising alternative agents for combating multidrug-resistant bacterial infections, including those caused by *K. pneumoniae* [151]. In recent years, several research groups have successfully isolated multiple phages capable of efficiently lysing *K. pneumoniae* from diverse environmental samples, and their specific characteristics are shown in Table 3. Features such as broad environmental adaptability, high lytic activity, and short latent periods support their potential as alternative antibiotics for controlling *K. pneumoniae* infections.

#### 9.3.1. Mechanism of Action of Bacteriophage Therapy Against *K. pneumoniae* in Bovine

The unique biological properties of phages enable their significant lytic activity against bovine mastitis-derived *K. pneumoniae*. The lytic activity of phages against bovine mastitis-derived *K. pneumoniae* is attributed to their unique biological properties. This activity supports two key strategies: phage–antibiotic synergy and cocktail therapy for MDR infections.

**Figure 5:**
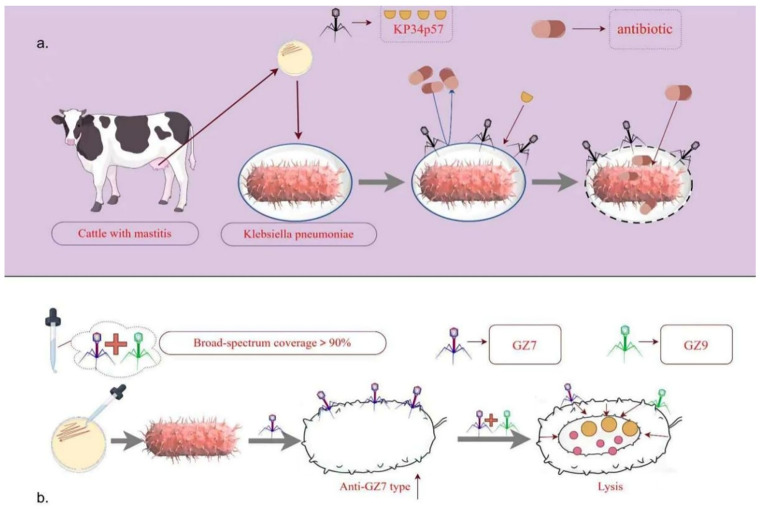
Phage–antibiotic synergy and phage cocktail therapy act against *K. pneumoniae* in bovine mastitis, effectively eliminating bacteria and reducing the risk of drug resistance. (**a**) Most depolymerases, encoded by phage tail fiber proteins, recognize exopolysaccharide receptors of *K. pneumoniae*, thereby clearing biofilms and rendering the bacteria more susceptible to antibiotics. (**b**) Compared with monophage treatment, two phages that target distinct receptors on *K. pneumoniae* exhibit enhanced antibacterial efficacy when applied simultaneously while reducing the emergence of resistant strains. Created with Figdraw.com. Author: [Yangseng Wang], (accessed on 13 November 2025).

Mechanism 1: Synergistic Phage–Antibiotic Therapy. This approach combines phages with antibiotics to produce a synergistic bactericidal effect while delaying or even reversing the development of resistance [153]. The underlying synergy operates through multiple mechanisms:

Biofilms are protective matrices formed by bacterial extracellular polymeric substances, which significantly increase bacterial tolerance to antibiotics. In synergistic therapy, phages directly disrupt the physical structure of biofilms through bacterial lysis, resulting in a looser architecture. This allows antibiotics, which would otherwise be largely excluded, to penetrate more deeply into the biofilm and effectively eliminate embedded bacteria. Specifically, the depolymerase Kp34p57 of *Klebsiella* phage Kp34 targets the KL64 capsule type and significantly inhibits biofilm formation [154,155]. A study revealed that combining phage HS106 with gentamicin reduced the MIC of gentamicin and led to significant clearance of mature biofilms [156]. A schematic of the mechanism is presented in Figure 5a.

Antibiotic-induced bacterial filamentation, as observed with β-lactams or SOS response-inducing agents in *E. coli*, increases the bacterial surface area, thereby enhancing phage adsorption and invasion. This accelerates the phage replication cycle and increases progeny yield [157].

The induction of a resensitization effect represents a key mechanism in phage–antibiotic synergy (PAS). Phage resistance in *K. pneumoniae* can come at the cost of restored antibiotic susceptibility. For example, strain Kp2092, through a mutation in the key phage receptor gene *galU*, exhibited increased antibiotic sensitivity [158]. Phages can also inhibit bacterial efflux pumps, promoting intracellular antibiotic accumulation and resensitizing *K. pneumoniae* to antibiotics [156]. This mechanism has also been documented in Salmonella [159] and Pseudomonas aeruginosa [160].

The second therapeutic approach involves phage cocktail therapy. Combining multiple phages enhances antibacterial efficacy and reduces the risk of resistance emergence. This strategy ensures broader coverage against target bacteria, thereby improving treatment success rates.

A phage cocktail with broad-spectrum activity (covering >90% of host strains) can effectively suppress *K. pneumoniae* growth [161]. A mixture of phages GZ7 and GZ9, for example, exhibited a host range covering 82.4% (42/51) of the tested strains. In vitro and in vivo studies confirmed that this cocktail significantly inhibits *K. pneumoniae* growth and reduces the emergence of phage-resistant mutants [162], positioning it as a promising solution for human *K. pneumoniae* infections [163]. The multitarget action of phage cocktails delays or suppresses the emergence of resistant mutants, and even if regrowth occurs, multiple targeting points help maintain control [164]. In vitro, the GZ7-GZ9 cocktail could eradicate GZ7-resistant bacteria [162]. A schematic of the mechanism is presented in Figure 5b.

Notably, phage resistance in *K. pneumoniae* can exert bidirectional or neutral effects on antibiotic susceptibility. For example, rpoN::Tn mutants presented increased susceptibility to polymyxins, whereas mutS::Tn and mutL::Tn mutants presented increased resistance to rifampicin and polymyxins [165]. These findings indicate that both synergistic and antagonistic effects may arise during combination therapy. Under phage pressure in vitro and in vivo, a CM8 mutant of the SCNJ1 strain developed resistance via a missense mutation in the *bglA* gene while maintaining virulence and fitness comparable to those of the wild-type strain in mice. This study is the first to report that *bglA* mutation confers phage resistance without an associated fitness cost [166]. Therefore, phage intervention does not invariably increase antibiotic sensitivity. Designing effective phage–antibiotic combinations requires a thorough dissection of the specific mechanisms behind phage resistance and an evaluation of their impact on antibacterial outcomes.

Owing to their efficient lytic activity against *K. pneumoniae*, phages—used synergistically with antibiotics or as cocktails—offer promising clinical strategies against multidrug-resistant infections, such as those caused by bovine mastitis-derived *K. pneumoniae*. Furthermore, the interplay between phage therapy, conventional treatments, and host immunity provides new perspectives for optimizing clinical strategies.

#### 9.3.2. Challenges in Bacteriophage Therapy

Bacteriophage therapy represents a promising alternative to antibiotics for bovine mastitis. However, its translation from the laboratory to the dairy farm faces several interconnected challenges across efficacy, safety, resources, and industrialization.

##### Challenges in Therapeutic Efficacy and Applicability

The extrapolation of efficacy from experimental models to clinical practice remains a primary hurdle. While promising results have been demonstrated in mouse mastitis models [75] and in vitro [88], their reproducibility in real-world farm settings and in large animals requires validation through robust, large-scale clinical trials. Furthermore, the host immune system can rapidly clear phages from circulation, potentially reducing their concentration at the infection site and diminishing therapeutic efficacy. Additionally, conventional phage therapy primarily targets extracellular bacteria. Effective treatment against intracellular pathogens occasionally involved in mastitis, such as Staphylococcus aureus and Salmonella Dublin [167,168], necessitates modified strategies like an engineered “Trojan horse” delivery system.

##### Limitations of Phage Resources and Bacterial Resistance

A critical barrier is the scarcity of well-characterized phages targeting specific bovine mastitis pathogens. Even for isolated phages, detailed information—including precise host ranges, bacterial receptors, phage resistance mechanisms, and co-evolution dynamics—is often lacking [167]. This makes it difficult to design rational phage cocktail formulations and to predict long-term therapeutic efficacy. This is particularly important given the capacity of bacteria to rapidly evolve resistance to phages (e.g., through receptor modification), a core challenge that underscores the need for precisely tailored combination therapies.

##### Hurdles in Safety, Production, and Regulatory Approval

Ensuring safety mandates stringent genomic analysis of therapeutic phages to confirm the absence of virulence or antibiotic resistance genes [169], a necessary but costly step in development. In addition to research and development, significant industrialization challenges exist. As “live biological products”, phages present complexities in large-scale standardized production, purification (especially endotoxin removal), and long-term stability. Most importantly, a clear regulatory approval pathway for veterinary phage products is largely lacking worldwide, which constitutes a major bottleneck for their commercial application.

### 9.4. Probiotics

A study showed that *Weissella cibaria SDS2.1* effectively inhibits the growth, adhesion, and invasion of *K. pneumoniae* in both in vitro and mouse mastitis models while significantly reducing peroxidase activity and the expression of inflammatory factors [170]. Similar in vitro effects have been observed with newly isolated bovine-derived strains, including *Lactobacillus paraplantarum SDN1.2* [171] and Enterococcus faecium MBBL3 [172]. These results suggest that probiotics hold promise as potential biological agents for assisting in the prevention or mitigation of *K. pneumoniae*-associated mastitis. However, validation in bovine mastitis models is still needed to assess their clinical potential.

### 9.5. Antimicrobial Peptides (AMPs)

In a whole-genome analysis of multiple mastitis pathogens, including *K. pneumoniae*, [173] screened key conserved core proteins (such as Rho and HupB) as targets and identified bacteriocin peptides (e.g., BP8) that can bind efficiently to these targets. Molecular dynamics simulations indicated that BP8 effectively inhibits bacterial replication and virulence. Integrating genomics with computational biology to identify pathogen targets represents a cutting-edge approach. However, clinical validation is still needed, and the technology remains costly.

## 10. Future Perspective

On the basis of existing research, the following perspectives are proposed for studies on *K. pneumoniae* associated with bovine mastitis:

First, *K. pneumoniae* strains that cause bovine mastitis exhibit regional epidemiological variation and virulence diversity. Genomic epidemiological surveys should be conducted, especially in regions with high incidence rates, and local transmission dynamics and virulence gene profiles should be integrated to inform the design of region-specific vaccines. Notably, carbapenem-resistant *K. pneumoniae* has formed a global epidemic pattern dominated by the production of KPC (especially KPC-2), NDM, and OXA-48 enzymes, with significant geographical differences. Moreover, carbapenem-resistant hypervirulent *K. pneumoniae*, particularly the ST11-KL64 lineage prevalent in China, spreads via mobile genetic elements such as plasmids and has become a major public health threat. Therefore, surveillance should focus on tracking the clonal spread of these drug-resistant and virulence-enhanced strains.

Second, compared with human-derived strains, hypermucoviscous strains derived from bovine mastitis exhibit substantial differences in virulence gene profiles. Although molecular diagnostic techniques such as whole-genome sequencing and AI-assisted MALDI-TOF MS enable rapid and accurate identification, their costs remain high. Thus, there is an urgent need to establish rapid detection technologies based on bovine-specific molecular markers.

Additionally, although mastitis caused by *K. pneumoniae* shares clinical manifestations with that caused by other pathogens, their pathogenic mechanisms—especially with respect to drug resistance—may differ: CRKP primarily employs mechanisms such as efflux pump overexpression, porin loss, and acquired carbapenemase production. The distribution and impact of these mechanisms in bovine-derived strains require systematic clarification.

Third, the current understanding of the mechanisms by which *K. pneumoniae* damages the blood-milk barrier during invasive infection is insufficient. Research has focused largely on the virulence factors of *K. pneumoniae* itself, but the response mechanisms of mammary epithelial cells during infection are poorly understood. Future studies could combine bovine mammary infection models with transcriptomic and proteomic approaches to systematically investigate the impact of *K. pneumoniae* on key physiological processes such as epithelial cell adhesion and cell death.

Moreover, the immune evasion mechanisms of *K. pneumoniae* in bovine mastitis are not yet clear. For example, whether it enhances adaptation within the mammary gland by modulating host defense proteins such as lactoferrin remains to be explored. Multi-omics analysis of host–pathogen interactions may help identify and validate key factors through which *K. pneumoniae* modulates the bovine immune response. With respect to treatment strategies, non-antibiotic approaches such as phage therapy (including phage–antibiotic synergy and cocktail formulations), probiotics (e.g., *Lactobacillus* and *Weissella cibaria SDS2.1*), and antimicrobial peptides (e.g., BP8) show potential. However, their synergistic mechanisms with antibaiotics, large-scale production, and regulatory policies remain current challenges.

Finally, from a “One Health” perspective, the environment (e.g., farm wastewater) serves as an important reservoir for the horizontal transfer of resistance genes via plasmids and may become a source of community- and animal-origin infections. Therefore, establishing a comprehensive drug resistance monitoring and management system spanning the human, animal, and environmental sectors is both urgent and of long-term importance for controlling the spread and evolution of *K. pneumoniae* in bovine mastitis.

## Figures and Tables

**Table 1:** Genotyping of *K. pneumoniae* from Global Bovine Mastitis.

Typing Method	Function	Types Identified	Countries	Sources	References
rep-PCR	Amplifies repetitive sequences to differentiate strains by banding patterns	rep-PCR types 1, 9, 17	USA, China	Farm environment, mastitis samples, and reinfection cases	[58,59,60,61]
RAPD	Uses random primers to assess DNA polymorphism and strain heterogeneity	RAPD type A	USA	Samples from mastitis outbreaks	[58]
PFGE	Separates large DNA fragments to distinguish genotypes	97 PFGE types (New York), 23 PFGE types (Wisconsin)	USA	Farm environment and mastitis samples	[57,59,62,63]
MLST	Sequences 7 housekeeping genes to assign sequence types (STs)	ST34, ST35, ST37, ST43, ST65, ST107, ST133, ST290, ST294, ST309, ST791, ST25, ST230, ST889, ST896, etc.	USA, China	Human and bovine isolates, mastitis samples	[32,34,57,59,64]
WGS-SNP	Analyzes genome-wide SNPs [59] to resolve phylogenetic groups	KpI, KpII, KpIII	Not specified	Clinical mastitis isolates	[14,18,22]

**Table 3:** Isolation sources and characteristics of *K. pneumoniae* phages from different environments.

Phage Name	Source	Morphotype	Optimal MOI	Latent Period (min)	Burst Size (PFU/Cell)	Stability	Reference
CM8–1	Waste water	Myoviridae	0.1	30	9.54	pH 6–10, 30–50 °C	[75]
vB_Kpn_B01	dairy farm trough	Siphoviridae	0.01	40	40 ± 3	pH 4–7, 37–50 °C	[152]

## Data Availability

No new data were created or analyzed in this study. Data sharing is not applicable to this article.
